# Incidence of Tracheal Stenosis in ICU Hospitalized COVID-19 Patients: Results from a Prospective, Observational, Multicenter Study

**DOI:** 10.3390/jpm14010039

**Published:** 2023-12-28

**Authors:** Gianluca Perroni, Dejan Radovanovic, Michele Mondoni, Giuseppe Mangiameli, Veronica Maria Giudici, Alessandro Crepaldi, Valentina Giatti, Emanuela Morenghi, Giulia Maria Stella, Stefano Pavesi, Marco Mantero, Angelo Guido Corsico, Maura Spotti, Chiara Premuda, Stefano Attilio Mangili, Elisa Franceschi, Veronica Macioce Narvena, Nicolò Vanoni, Tommaso Pilocane, Gianluca Russo, Fabiano Di Marco, Marco Alloisio, Stefano Aliberti, Giuseppe Marulli, Alexia Francesca Bertuzzi, Giuseppe Cipolla, Stefano Centanni, Francesco Blasi, Pierachille Santus, Umberto Cariboni

**Affiliations:** 1 Division of Thoracic Surgery, IRCCS Humanitas Research Hospital, Rozzano, 20089 Milan, Italy; gianlucaperroni91@gmail.com (G.P.); veronica.giudici@cancercenter.humanitas.it (V.M.G.); alessandro.crepaldi@humanitas.it (A.C.); valentina.giatti@humanitas.it (V.G.); marco.alloisio@hunimed.eu (M.A.); giuseppe.marulli@hunimed.eu (G.M.); umberto.cariboni@humanitas.it (U.C.); 2 Division of Respiratory Diseases, L. Sacco University Hospital, ASST Fatebenefratelli-Sacco, Department of Biomedical and Clinical Sciences, Università Degli Studi di Milano, 20157 Milan, Italy; dejan.radovanovic@unimi.it (D.R.); elisa.franceschi@unimi.it (E.F.); stefano.centanni@unimi.it (S.C.); pierachille.santus@unimi.it (P.S.); 3 Respiratory Unit, ASST Santi Paolo e Carlo, Department of Health Sciences, University of Milan, 20122 Milan, Italy; michele.mondoni@asst-santipaolocarlo.it (M.M.); stefano.pavesi@asst-santipaolocarlo.it (S.P.); 4 Department of Biomedical Sciences, Humanitas University, 20072 Milan, Italy; stefano.aliberti@hunimed.eu; 5 Biostatistics Unit, IRCCS Humanitas Research Hospital, 20089 Milan, Italy; emanuela.morenghi@humanitas.it; 6 Department of Internal Medicine and Medical Therapeutics, IRCCS Policlinico San Matteo Foundation, 27100 Pavia, Italy; g.stella@smatteo.pv.it (G.M.S.); angelo.corsico@unipv.it (A.G.C.); 7 Unit of Respiratory Diseases, Cardio-Thoraco-Vascular Department, IRCCS Policlinico San Matteo Foundation, 27100 Pavia, Italy; 8 Respiratory Unit and Cystic Fibrosis Adult Center, Fondazione IRCCS Ca’ Granda Ospedale Maggiore Policlinico, 20122 Milan, Italy; marco.mantero@unimi.it (M.M.); maura.spotti@policlinico.mi.it (M.S.); tommaso.pilocane@unimi.it (T.P.); francesco.blasi@unimi.it (F.B.); 9 Department of Pathophysiology and Transplantation, University of Milan, 20122 Milan, Italy; 10 Otolarygoiatry Unit, Sant’anna Istitute, 25127 Brescia, Italy; stefano.mangili@gmail.com; 11 Unit of Pneumology of Codogno, ASST Lodi, 26845 Lodi, Italy; veronica.narvena@hotmail.com; 12 Unit of Pneumology, ASST Lodi, 26900 Lodi, Italy; nicolo.vanoni@asst-lodi.it (N.V.); giuseppe.cipolla@asst-lodi.it (G.C.); 13 Unit of Pain Medicine, Department of Emergency, ASST Lodi, 26900 Lodi, Italy; gianluca.russo@asst-lodi.it; 14 Department of Health Sciences, Università degli Studi di Milano, Pneumologia, ASST Papa Giovanni XXIII, 24127 Bergamo, Italy; fabiano.dimarco@unimi.it; 15 Unit of Pneumology, IRCCS Humanitas Research Hospital, 20089 Milan, Italy; 16 Department of Oncology & Hematology, IRCCS Humanitas Research Hospital, 20089 Milan, Italy; alexia.bertuzzi@cancercenter.humanitas.it

**Keywords:** COVID-related tracheal stenosis, SARS-CoV-2 pandemic, tracheal stenosis, tracheostomy

## Abstract

**Background:** Tracheal stenosis represents a fearsome complication that substantially impairs quality of life. The recent SARS-CoV-2 pandemic increased the number of patients requiring invasive ventilation through prolonged intubation or tracheostomy, increasing the risk of tracheal stenosis. **Study design and methods:** In this prospective, observational, multicenter study performed in Lombardy (Italy), we have exanimated 281 patients who underwent prolonged intubation (more than 7 days) or tracheostomy for severe COVID-19. Patients underwent CT scan and spirometry 2 months after hospital discharge and a subsequent clinical follow-up after an additional 6 months (overall 8 months of follow-up duration) to detect any tracheal lumen reduction above 1%. The last follow-up evaluation was completed on 31 August 2022. **Results:** In the study period, 24 patients (8.5%, CI 5.6–12.4) developed tracheal stenosis in a median time of 112 days and within a period of 200 days from intubation. Compared to patients without tracheal stenosis, tracheostomy was performed more frequently in patients that developed stenosis (75% vs 54%, *p* = 0.034). Tracheostomy and alcohol consumption (1 unit of alcohol per day) increased risk of developing tracheal stenosis of 2.6-fold (*p* = 0.047; IC 0.99–6.8) and 5.4-fold (*p* = 0.002; CI 1.9–16), respectively. **Conclusions**: In a large cohort of patients, the incidence of tracheal stenosis increased during pandemic, probably related to the increased use of prolonged intubation. Patients with histories of prolonged intubation should be monitored for at least 200 days from invasive ventilation in order to detect tracheal stenosis at early stage. Alcohol use and tracheostomy are risk factors for developing tracheal stenosis.

## 1. Introduction

Tracheal stenosis represents a serious and potentially life-threatening complication following prolonged intubation or tracheostomy. It is a relatively rare event, with an incidence of 1/200,000 people per year, with symptoms typically arising after three to four weeks after removal of tracheal tube or tracheostomy [1]. Since the onset of the SARS-CoV-2 pandemic, an increased number of patients with tracheal stenosis have been referred to centers specialized in advanced surgical airways treatment [2], mostly from geographical areas with high incidences of COVID-19, such as the Lombardy region in Italy [3].

Several factors might have contributed in determining the occurrence of tracheal stenosis: the large number of critically ill patients who required endotracheal intubation, the prolonged exposure to mechanical ventilation, the high rate of weaning failures and consequent re-intubations, the large number of tracheostomies performed and the inflammatory state of the upper airways associated with the SARS-CoV-2 infection [3].

Indeed, SARS-CoV-2 replicates not only in pneumocytes but also in the tracheal epithelium, causing inflammation and chronic tracheitis that can potentially weaken the mucosa [4]. Furthermore, the prothrombotic state and the fibrinolysis shutdown related to COVID-19 are the main causes of microvascular injuries [5,6] that affect many organs, including the tracheal epithelium.

Treatment of disease may also contribute to tracheal damage. Pronation of patients during mechanical ventilation can lead to an increase in cuff pressure and to dislocation of the endotracheal tube, thus causing direct mechanical damage. Re-intubations, which have often been necessary in patients with COVID-19, represented a source of damage for the tracheal epithelium [7,8]. Finally, pharmacological treatment based on corticosteroids may impair normal mucosal healing [9].

The knowledge of the main risk factors responsible for tracheal stenosis associated with COVID-19 infection is poor and often derived from retrospective studies. No studies have prospectively investigated the incidence of tracheal stenosis in a large cohort of patients requiring prolonged intubation and COVID-19. In this prospective, observational, multicenter Italian study, we aimed to assess the incidence of tracheal stenosis and identify the main risk factors for the occurrence of tracheal stenosis related to intubated patients with COVID-19.

## 2. Methods

### 2.1. Design and Setting

This was a prospective, observational, multicenter study performed in seven Italian hospitals located in Lombardy (three academic hospitals and four non-academic tertiary hospitals) from 1 January 2020 to 31 December 2021. We consecutively enrolled adult patients (older than 18 years of age) that underwent prolonged intubation (defined as a permanence of naso- or oro-tracheal tube for more than 7 days [10]) or tracheostomy (surgical or percutaneous) for respiratory failure due to microbiologically confirmed SARS-CoV-2 infection (i.e., with positive polymerase chain reaction on nasopharyngeal swab or bronchoalveolar lavage). For each patient, data on demographic characteristics; smoking or alcohol habits; and past medical history, including comorbidities, prior history of prolonged ventilation or tracheotomy, duration of intensive care unit (ICU) stay, and treatment were collected. Alcohol consumption was estimated per patient by adopting alcohol units. A unit of alcohol was calculated using the following formula: [Volume (ml) × Alcohol by Volume ABV (%)]/1000.

The enrolled cohort was followed-up for at least 2 months and for up to 8 months after being discharged from the ICU. During the follow-up, the following information was collected: occurrence of tracheal stenosis and timing of occurrence (days) calculated from the hospital discharge. IRCCS Humanitas Research Hospital (Rozzano, Italy) was the academic hospital defined as the referral center for specialist surgical evaluation aimed at confirming and managing any tracheal stenosis detected during the follow-up period.

### 2.2. Follow-Up Strategies

All patients were offered an initial evaluation according to clinical practice, including a non-contrast computed tomography (CT) scan of the neck and the chest within 2 months from hospital discharge and pulmonary function testing (PFT) using standard spirometry [11]. The latter was employed to calculate the expiratory disproportion index (EDI), i.e., the ratio of forced expiratory volume in 1 s (FEVI) and peak expiratory flow rate (PEFR), according to the following formula: (EDI = FEVI[L]/PEFR[L/s] × 100). EDI was employed to differentiate upper airway stenosis from other etiologies of dyspnea [12]. Tracheal stenosis was classified according to the system of Cotton and Myer [13]. In case of discordance between CT and EDI score (e.g., tracheal stenosis < 30% at CT scan with EDI score > 50), priority was assigned to the CT result.

Patients with radiological evidence of tracheal stenosis > 30% or with an EDI score > 50 or symptoms of obstruction (i.e., dyspnea, wheezing, cough) were immediately referred for a bronchoscopic and surgical evaluation at the referral center and treated according to good clinical practice.

Patients with tracheal stenosis ≤ 30% at CT scan or EDI score ≤ 50 without symptoms of obstruction at the initial examination underwent clinical and functional follow-up 3 and 6 months after. If the patient developed symptoms of tracheal stenosis at any time, they would undergo a CT scan and PFT as described above. Patients with no clinical, functional, or radiological signs of tracheal stenosis at initial evaluation underwent clinical follow-up 3 and 6 months after; CT and PFE were performed in case of development of symptoms suggestive of tracheal stenosis at any time. During follow-up, the Airway-Dyspnoea-Voice-Swallow (ADVS) questionnaire was administered 2, 5 and 8 months after hospital discharge [12].

### 2.3. Study Endpoints

The primary endpoint of the study was to determine the incidence of tracheal stenosis in critically ill COVID-19 survivors exposed to prolonged intubation or tracheostomy. Secondary endpoints were the identification of risk factors implicated in the occurrence of tracheal stenosis and of the time of occurrence of tracheal stenosis.

### 2.4. Ethical Review of the Study

This study was conducted in accordance with the Declaration of Helsinki (Tokyo, Venice, Hong Kong, and Somerset West amendments) and Italian laws and regulations. The protocol was drafted and the study conducted according to the ICH Guidelines for Good Clinical Practice (GCP). The protocol and its annexes were reviewed and approved by the relevant Independent Ethics Committee (IEC, code TS1) of IRCCS Humanitas Research Hospital (Milan, Italy) and by the ethics committees of each participating center. The study was registered at clinicaltrials.gov (accessed on 20 December 2023) (NCT04686721). Informed consent was obtained for all patients at hospital admission or at the time of discharge.

### 2.5. Statistical Analysis

The study enrolled all patients with inclusion/exclusion criteria in a 6-month period, forecasting a final sample size of approximately 200 patients, allowing a standard error of the prevalence of tracheal stenosis in COVID-19 patients with prolonged intubation or tracheostomy—expected to be between 20 and 25%—of no more than 3%.

Data were collected and analyzed using the web-based platform Research Electronic Data Capture (REDcap, version 13.4.12) and Stata version 17 (StataCorp. 2021. Stata Statistical Software: Release 17. College Station, TX: StataCorp LLC), respectively. Data were described as number, percentage, and 95% confidence interval for the primary endpoint. All other variables were described as number and percentage if categorical; mean and standard deviation if continuous; and Gaussian distribution or median and range if not normally distributed. Adherence to the Gaussian distribution was checked with Shapiro’s test. The association of stenosis with other variables was checked with the chi-square test if categorical or with the t-Student or Mann–Whitney test if continuous, as appropriate. The association with the presence of tracheal stenosis was explored with logistic regression analysis. 

Time to stenosis was defined as the time between admission and first stenosis or last contact date. Survival from stenosis was described using the Kaplan–Meier method. Association of possible risk factors was explored with a proportional hazard Cox regression. Due to the relatively small number of events, no multivariable analysis was performed. A *p*-value < 0.05 was considered to be significant. 

## 3. Results

### 3.1. Patients’ Characteristics

During the study period, a total of 281 patients met the inclusion criteria and were enrolled in the study. Anthropometric and clinical characteristics of all patients are summarized in Table 1. Patients were mostly male (*n* = 225; 80%), with a mean (SD) age of 61 (±10) years; 63% of patients were non-smokers, while 161 had diabetes (57%); 22 patients (9%) had chronic obstructive pulmonary disease (COPD), and 21 (7.5%) had gastroesophageal reflux disease (GERD); 20 patients (7.1%) consumed alcohol on a daily basis. Body mass index (BMI) was 29 (±6) Kg/m^2^, and the mean intubation time was 23 (±16) days. Starting from the day of intubation, a total of 27 patients (9.6%) died. 

### 3.2. Follow-Up Period

**Figure 1:**
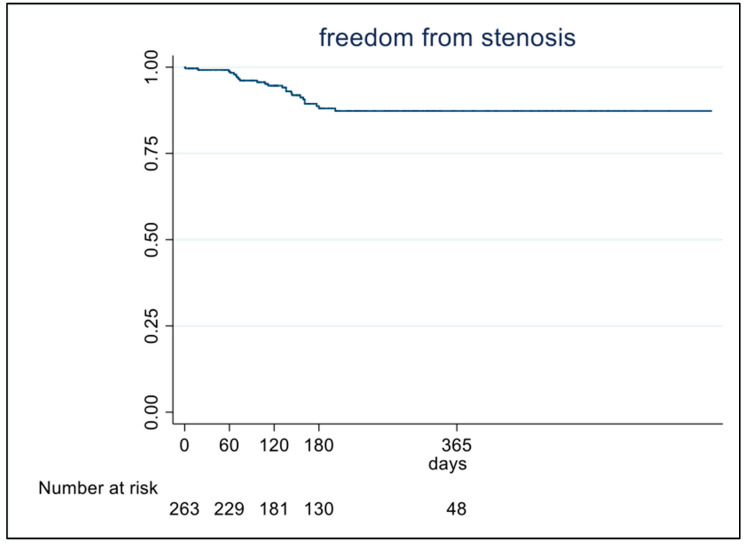
Occurrence of tracheal stenosis during follow-up period in the overall population.

During the follow-up period, 24 patients developed tracheal stenosis, with a prevalence of 8.54% (95%CI: 5.6–12) and an incidence rate of 0.012 patients/month (95% CI 0.0074–0.018). The median (range) duration of follow-up was 6 months (0–23 months). Grade I tracheal stenosis was observed in 71% of cases, while grade III and IV stenosis accounted for 13% of the total cases of our cohort (Table 1). The median time to stenosis was 112 days. The latest case of tracheal stenosis was identified 200 days after orotracheal intubation (Figure 1).

Comparing the clinical characteristics of patients who developed stenosis to non-stenotic patients, the only difference was represented by alcohol consumption (Table 2). Alcohol consumption was significantly more frequent in patients with stenosis (25% vs. 5.8%; *p* = 0.002), and the number of alcoholic units were also higher in this group (0.75 ± 1.7 vs. 0.10 ± 0.57; *p* = 0.005) compared to non-stenotic patients.

**Figure 2:**
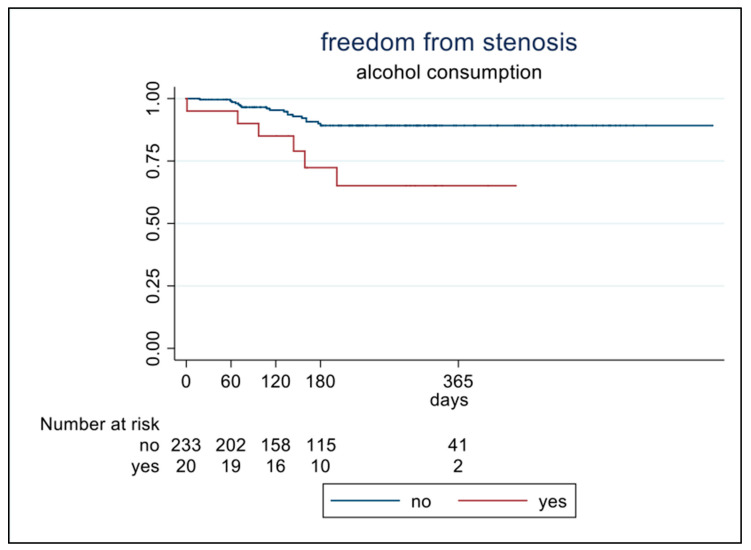
Patients stratified according to alcohol consumption.

At univariate analysis, alcohol consumption increased the risk of developing tracheal stenosis 5.4-fold (*p* = 0.002; CI 1.9–16) (Figure 2). Tracheostomy was more frequently performed in patients that developed tracheal stenosis (75% vs. 54%, *p* = 0.034) and increased the risk of tracheal stenosis 2.6-fold (*p* = 0.047; IC 0.99–6.8) at univariate analysis (Table 3). EDI value was significantly higher in patients with stenosis (45 ± 13 vs. 39 ± 10; *p* = 0.025), whereas the most frequent symptoms experienced by patients with stenosis were dyspnea and voice changes (Table 4).

## 4. Discussion

As a result of the COVID pandemic, a greater number of tracheal stenosis cases have been diagnosed and managed both in peripherical and referral airway centers over the last two years.

This is, to our knowledge, the first prospective multicenter study assessing the incidence of tracheal stenosis in a cohort of patients with COVID-19 admitted to ICU who underwent either prolonged intubation or tracheostomy. We first demonstrated, in a large cohort of patients, a prevalence of stenosis of 8.5% and an incidence of 14 cases/100 person-year.

Our findings confirm the recognized dramatic increase in tracheal stenosis incidence as a consequence of an increased use of invasive ventilation during the SARS-CoV-2 pandemic [2]. 

The latest tracheal stenosis was identified 200 days after orotracheal intubation. This is a key finding with important entailment in deciding the optimal duration of follow-up in patients with suspected tracheal stenosis. The reason for this delay is unclear.

Based on our findings, alcohol is a risk factor for stenosis of large airways damaged by prolonged intubation. Indeed, alcohol use and the number of alcohol units are significantly more prevalent in patients with stenosis in our study. Alcohol use is related to many deleterious effect on respiratory epithelium, with several mechanisms of actions: alcohol is a highly volatile substance and—unlike oxygen, which is exchanged at the alveolus–capillary interface—its diffusion into the respiratory system occurs from the bronchial circulation through the ciliated epithelium [14]. In addition, vaporized alcohol can be deposited back into the respiratory tree causing a “recirculation” phenomenon that exposes the respiratory epithelium to high concentrations of alcohol for a prolonged period of time [14]. Alcohol has a dual effect on ciliary motility: despite an initial increase in ciliary beat, it then leads to induction of alcohol-sensitive phosphodiesterase and downregulation of protein kinase A, with subsequent reduction in ciliary function [15]. Alcohol impairs the normal apposition of granulation tissue; it delays the normal skin repair cycle, as it promotes an exaggerated inflammatory response [16].

When the initial danger of injury is contained, pro-inflammatory signaling molecules decrease. There is an increase in regulatory T cells (Tregs) and an increase in IL-10 and TGF-β, creating an anti-inflammatory microenvironment that allows the predominant macrophage population to adopt a wound-healing phenotype. Thus, we hypothesize that alcohol damage could be an effect of upregulating the TGF-β signaling pathway.

In our study, smoking tobacco was not associated with stenosis development. Although a smoking habit is a simple condition to evaluate, we have found only one study in the literature that reaches opposite conclusions to ours. In this series, smoking tobacco is a risk factor for developing tracheal stenosis, and its effect may be due to the established detrimental effects of tobacco on wound healing, including decreased vascularization [17]. On the other side of the literature, tobacco-smoking seems to be a well-defined risk factor that should be considered when tracheal stenosis treatments are adopted. In particular, in a series of 119 patients affected by central tracheal stenosis submitted to several attempts of interventional therapeutic bronchoscopy, smoker status was an independent factor associated with reduced long-term efficacy of interventional bronchoscopy. The authors concluded that smoking cessation should be encouraged to improve the outcome of therapeutic bronchoscopy [18]. Similarly, in a series of 34 patients who underwent tracheal resection and anastomosis in postintubation tracheal stenosis, smoking was significantly associated with re-stenosis after a surgical treatment [19].

Notably, no differences in the duration of intubation between patients with and without stenosis were recorded, while tracheotomy was performed more frequently in the first group (75% vs. 52%, *p* = 0.03). Tracheostomy represents a well-known risk factor for stenosis. Similar to our findings, a recent study showed that tracheostomy was performed more frequently in COVID-19 patients and represented an additional risk factor for stenosis in this group of patients [20].

The exact role of tracheostomy in its association with tracheal stenosis remains unclear, even if it is suspected to be related to epithelial damage. We hypothesize that mechanical trauma related to tracheostomy and tracheal cannula may represent the main mechanism of action, followed by abnormal wound healing with development of granulomas; additionally, cartilage damage may also contribute to granuloma formation [21]. The type of tracheostomy (surgical or percutaneous) appears to be unrelated to an increased tendency to stenosis. Several studies report conflicting findings concluding that the choice of technique should not be based on the potential risk of tracheal stenosis [22]. 

In a recent study on the surgical treatment of tracheal stenosis during COVID-19 pandemic, a large proportion of tracheostomy procedures was associated with complications such as high tracheostomy placement (just below cricoid or in the first tracheal ring), broken tracheal rings, and/or inappropriate airway sites. We speculate that the high rate of complications was associated with the high frequency of emergency tracheostomy during the pandemic [20]. Almost all patients who developed tracheal stenosis in this study underwent surgery. We have recently reported COVID tracheal stenoses as being more likely to be treated with surgery compared to non-COVID considering their location—usually more distal from the vocal folds—and their trend to involve a greater number of tracheal rings [20]. Several recent surgical series confirm this evidence [23,24,25,26,27,28].

Interestingly, the duration of orotracheal intubation was similar in the two groups in our study. Recent evidence showed a median duration of invasive ventilation of about 17 days. In our series, this time was higher (about 30 days), and this supports our data concerning the role of tracheostomy as a risk factor for developing tracheal stenosis independent of the time of exposure to orotracheal intubation. In fact, it has been demonstrated that the risk of developing tracheal stenosis increased by 20% per day of intubation regardless of COVID-19 severity [20].

Finally, in our study, EDI was confirmed as a good tool that can help differentiate upper airway stenosis from other dyspnea etiologies. Dyspnea together with voice changes was the most common symptom reported during follow-up [29,30].

Another advantage of ED is probably to overcome the considerable diagnostic delay usually reported in the diagnosis of benign acquired subglottic stenosis in adults. Patients are frequently misdiagnosed because symptoms of this disease may mimic symptoms of asthma. The Expiratory Disproportion Index (EDI) obtained via spirometry may be a simple instrument to detect a tracheal stenosis in symptomatic patients early in a post-pandemic scenario.

### Study Limitations

Our study has a number of limitations. First, the exact impact of pharmacological treatments (e.g., corticosteroid and/or antiviral drugs) administered to treat COVID-19 on inducing tracheal stenosis has not been investigated. Second, the role of the prone position in determining the occurrence of stenosis in intubated COVID-19 patients was not assessed. The strengths of the study include its prospective design, the homogenous characteristics of our cohort in the time of intubation, the time to tracheostomy, and the long-term follow-up. With the aim to homogenize the study population and correctly define the frequency of tracheal stenosis and the timing of occurrence, we have exclusively included patients respecting the abovementioned inclusion criteria (prolonged intubation for more than 7 days or tracheostomy). The proportion of patients lost during follow-up, included 27 patients who died, probably contributes to underestimating the frequency of tracheal stenosis reported in our study.

## 5. Conclusions

In conclusion, we describe in this multicenter prospective study a prevalence and incidence of tracheal stenosis of respectively 8.5% (14 cases/100 person-year) among patients undergoing prolonged intubation (with or without tracheotomy) for COVID-19. All tracheal stenoses were identified within 200 days after orotracheal intubation, suggesting an optimal follow-up duration of patients at risk for tracheal stenosis of at least 8 months. Both alcohol consumption and tracheostomy increase the risk of developing tracheal stenosis, but further studies in animal models are warranted in elucidating the exact mechanism of action. EDI may be a useful, non-invasive diagnostic tool to differentiate upper airway stenosis from other dyspnea etiologies.

## Figures and Tables

**Table 1:** Main demographic and clinical characteristics of the study cohort. Variables are expressed as frequencies (percentage) and mean (standard deviation) as appropriate.

		Patients (n = 281)
Age, Years		61 (±10)
Gender (Male), *n* (%)		225 (80%)
Smoking status, *n* (%)	Never	176 (63%)
	Former	65 (23%)
	Current	28 (10%)
	N/A	12 (4.3%)
Alcohol use, *n* (%)	No	245 (87%)
	Yes	21 (7.5%)
	N/A	15 (5.5%)
GERD, *n* (%)		21 (7.5%)
Diabetes, *n* (%)	No	161 (57%)
	Type I	62 (22%)
	Type II	56 (20%)
	N/A	2 (0.7%)
COPD/Asthma, *n* (%)		44 (16%)
Stenosis, *n* (%)		24 (8.5%)
Stenosis grade, *n* (%)	I	17 (71%)
	II	4 (17%)
	III	1 (4%)
	IV	2 (8%)

GERD: gastroesophageal reflux disease; COPD: chronic obstructive pulmonary disease.

**Table 2:** Demographic and clinical characteristics of the cohort related to the presence of tracheal stenosis.

	Stenosis (n = 24)	No Stenosis (n = 257)	OR (95%CI)	*p*-Value
Male sex, *n* (%)	18 (75%)	207 (81%)	0.72 (0.27–1.9)	0.53
Age (mean, SD)	61 ± 9.7	62 ± 11	1.01 (0.97–1.1)	0.67
BMI (mean, SD)	27.7 ± 5.1	29.7 ± 6.1	1.07 (0.99–1.1)	0.074
Smoking status (*n* = 245)				
Never, *n* (%)	16 (67%)	160 (65%)	1	
Former, *n* (%)	7 (29%)	58 (24%)	1.2 (0.47–3.1)	0.69
Current, *n* (%)	1 (4.2%)	27 (11%)	0.37 (0.05–2.9)	0.35
Alcohol consumer, *n* (%)	6 (25%)	14 (5.8%)	5.4 (1.9–16)	0.002
Alcohol units (mean, SD)	0.75 ± 1.7	0.10 ± 0.57	1.7 (1.2–2.5)	0.005
GERD, *n* (%)	1 (4.2%)	20/255 (7.9%)	0.51 (0.07–4.0)	0.52
Diabetes (*n* = 255)				
No, *n* (%)	12 (50%)	149 (58%)	1	
Type I, *n* (%)	3 (13%)	59 (23%)	0.63 (0.17–2.3)	0.49
Type II, *n* (%)	9 (38%)	47 (18%)	2.4 (0.94–6.0)	0.066
COPD, *n* (%)	2 (8.3%)	22/255 (8.6%)	0.96 (0.21–4.4)	0.96
Asthma, *n* (%)	2 (8.3%)	18/255 (7.1%)	1.2 (0.26–5.5)	0.82

BMI: body mass index; GERD: gastroesophageal reflux disease; COPD: chronic obstructive pulmonary disease.

**Table 3:** Main characteristics of intubation and tracheostomy related to the presence of tracheal stenosis.

	Stenosis (*n* = 24)	No Stenosis (*n* = 257)	*p*-Value
Days from admission to intubation (median, range)	2 (0–9)	2 (0–366)	0.689
Days of intubation (median, range)	24 ± 16	23 ± 17	0.527
Tracheostomy performed (count, percentage)	18 (75%)	134 (52%)	0.034
Days of tracheostomy (median, range)	34 (5–168)	29 (4–126)	0.125

**Table 4:** Data collected during follow-up.

	Stenosis (*n* = 24)	No Stenosis (*n* = 257)	*p*-Value
EDI	45 ± 13 (*n* = 18)	39 ± 9.9 (*n* = 142)	0.025
Dyspnea	18/31 (58%)	107/377 (28%)	0.001
Voice change	9/30 (30%)	38/377 (10%)	0.001

## Data Availability

The data that support the findings of this study are available on request from the corresponding author.

## Abbreviations

ADVS	Airway-Dyspnea-Voice-Swallow
CT	computed tomography
EDI	expiratory disproportion index
FEVI	forced expiratory volume in 1 s
ICU	intensive care unit
PEFR	peak expiratory flow rate
PFT	pulmonary function testing
